# The complete mitochondrial genome of *Calidris tenuirostris* (Charadriiformes: Scolopacidae)

**DOI:** 10.1080/23802359.2019.1698375

**Published:** 2019-12-12

**Authors:** Yuxiao He, Wei Liu, Yanqing Wu, Qing Chang

**Affiliations:** a Ministry of Ecology and Environment of the People's Republic of China, Nanjing Institute of Environmental Sciences, Jiangsu, People’s Republic of China;; b Jiangsu Key Laboratory for Biodiversity and Biotechnology, College of Life Sciences, Nanjing Normal University, Jiangsu, People’s Republic of China Nanjing

**Keywords:** *Calidris tenuirostris*, mitochondrial genome, phylogenetic analysis

## Abstract

There is a lack of genomic information on *Calidris tenuirostris*, which can provide valuable resource for genetic diversity and conservation biology. The complete mitogenome of *C. tenuirostris* was 16,775 bp in length and contained 13 protein-coding genes (PCGs), 2 ribosomal RNA (rRNA) genes, 22 transfer RNA (tRNA) genes and 1 control region (D-loop). The overall nucleotide composition was 31.10% A, 24.9% T, 30.10% C and 13.9% G. The start codon (ATG) and end codon (TAA) were usual. Phylogenomic analysis using mitogenomes supports monophyly of Scolopacidae, and help us understand the evolutionary relationship in charadriiformes.

Great knot *Calidris tenuirostris* is (Charadriiformes, Scolopacidae) is the largest species in *Calidris* genus with long distance migration, which breeding on tundra in northeast Siberia and wintering on the coasts from Southern Asia through to Australia (Hua et al. 2017). Although there are numerous individuals in the East Asian-Australasian Flyway with an estimated population size of 290,000, the population of great knot is declining rapidly under the multiple environmental stress (Melville et al. 2016; Studds et al. 2017). And, this species has been listed in the IUCN Red List of Endangered Species and has also been considered to be the indicator of the quality of coastal wetland (BirdLife International 2016). Recently, many studies focus on the habitat ecology, migratory phenology and behavior to try to explain the terrible situation (Peng et al. 2015; Tan et al. 2018). However, few studies attempted to reveal why some species from Charadriiformes are endangered by analyzing the genetic feature (Liu et al. 2018; Subramanian 2018). Thus, it is necessary to figure out the genetic structure and phylogenetic relationship, which may facilitate understand the fitness and selective pressure of great knot.

In this study, the complete mitochondrial genome sequence of *C. tenuirostris* was determined and deposited into GenBank (GenBank accession number: MK992912). The samples were collected from Rudong, Jiangsu Province, China, in the autumn migratory season in 2017 by mist net and cannon net (121°7′58.06″E, 32°31′55.60″N). Feather and blood sample was preserved with 90% ethanol in sterilized centrifuge tube and stored at –80 °C in the Nanjing Normal University (specimen voucher number: NJNU- Calt02). The genomic DNA was isolated by the standard phenol–chloroform extraction procedure (Sambrook and Russell 1989).

Sequences obtained were assembled and edited using the SeqMan (DNAstar, Inc) to generate the complete mitochondrial genome. The complete mitochondrial genome of *C. tenuirostris* was a circular molecular with 16,775 bp in total length, containing 13 protein coding genes, two ribosomal RNAs genes, 22 transfer RNAs genes and a non-coding control region (D-loop). The overall base composition was 31.10% A, 24.9% T, 30.10% C and 13.9% G.

Phylogenetic analysis based on the complete mitochondrial genome was conducted using Bayesian (BI) method and Maximum-likelihood (ML) method. The result of BI and ML was consensus, and indicated that *C. tenuirostris* showed a close relationship with *Eurynorhynchus pygmeus* and *Arenaria interpres* (Figure 1), which mean these three genus might own a same ancestor. The study improves our understanding of mitogenome structure and evolution in Charadriiformes.

**Figure 1.**
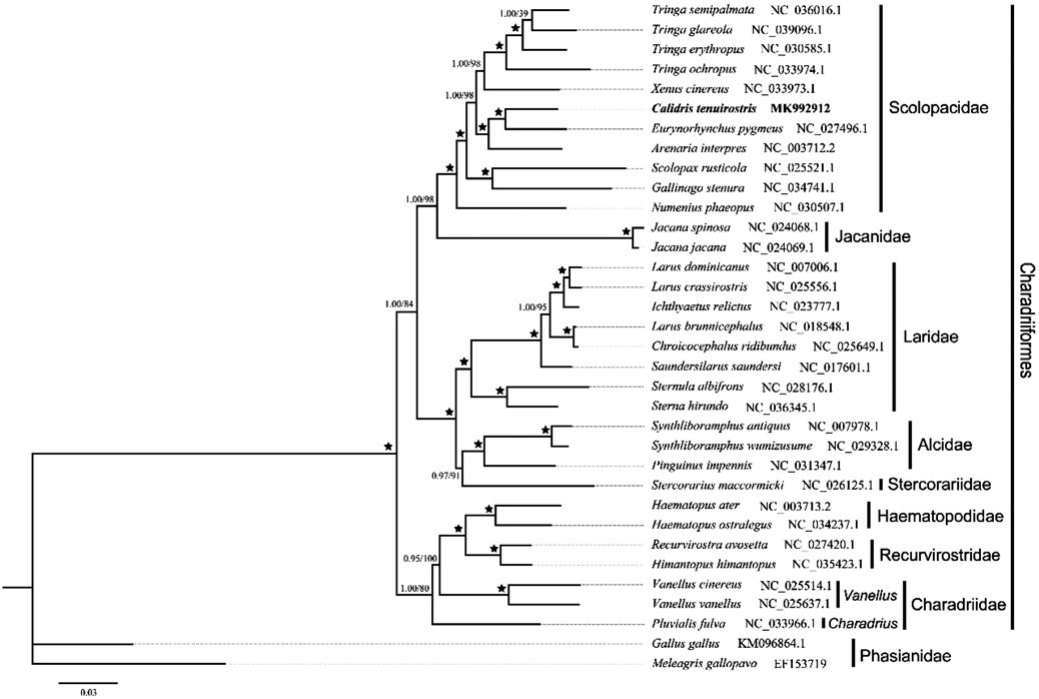
The molecular phylogenomic analysis of *Calidris tenuirostris* within Charadriiformes based on the complete mitochondrial genome. Stars indicated the nodes received a posterior probability of 1.00 and 100 bootstrape value.
